# Supplementary material for: Condensation of LINE-1 is critical for retrotransposition
Source: eLife. 2023 Apr 28;12:e82991. doi: 10.7554/eLife.82991 (PMC10202459; doi:10.7554/eLife.82991)
Supplement: Figure 1—source data 1. — Output data matrix from GEMspa with tracking data for all tracked ORF1 particle trajectories in HeLa cells expressing the reporter L1 for 24 hr; associated with Figure 1D. [file elife-82991-fig1-data1.zip › Figure 1-Source Data 1/Figure 1-Source Data 1 README.docx]

Figure 1-Source Data 1

ORF1punctaTracking_GEMspa_NucCyto_all_data.csv

- Output data matrix from GEMspa with tracking data for all tracked ORF1 particle trajectories, with the following columns:
  - id: movie identification number
  - file name: movie file name
  - location: Cytoplasm or Nucleus, based on input image masks for nuclei
  - roi: If a mask image was used to identify separate cells, then the label of the region is listed here.
  - group: analysis group (Cytoplasm vs Nucleus)
  - D_median: median Deff over all tracks in the file/ROI
  - D_mean: mean Deff (as above)
  - D_median_filt: median Deff over all tracks where min <= Deff <= max from Run parameters; in this case, min=0.005 but the filtered data was not used for analysis
  - D_mean_filt: mean filtered Deff (as above)
  - Trajectory: trajectory identification number
  - int_mean: mean intensity of the particle
  - int_std: standard deviation of the particle intensity
  - D: Deff for the given track/trajectory
  - err: one standard deviation error on the estimated parameter (D)
  - r_sq: r^2^ for the fit
  - rmse: root mean square error (residuals)
  - track_len: the length of the given track/trajectory
  - D_max_tlag: max tlag considered for fitting the diffusion equation
