# Supplementary material for: Condensation of LINE-1 is critical for retrotransposition
Source: eLife. 2023 Apr 28;12:e82991. doi: 10.7554/eLife.82991 (PMC10202459; doi:10.7554/eLife.82991)
Supplement: Figure 1—source data 2. — Output data matrices from the spot detection algorithm run on the ORF1-Halo and ORF1-mNG2 colocalization data, with intensity information for the detected intracellular JF549+ spots and randomly assigned intracellular spots; associated with Figure 1F. [file elife-82991-fig1-data2.zip › Figure 1-Source Data 2/Figure 1-Source Data 2 README.docx]

Figure 1-Source Data 2

ORF1-561-all_ORF1_spots-NORM.csv

- Output data matrix from the spot detection algorithm run on the ORF1-Halo and ORF1-mNG2 colocalization data, with intensity information for the detected intracellular JF549+ spots, including intensities in the JF549 channel, the JF646 channel, and the mNG2 channel, with the following columns:
  - plane (z): Z plane of the detected spot
  - row (y): y coordinate of the detected spot
  - col (x): x coordinate of the detected spot
  - radius: radius of the detected spot
  - roi: image name or ROI name in which the spot was detected
  - nuclei_ch_intensity: DAPI intensity at the detected spot
  - JF549_intensity: raw JF549 intensity at the detected spot
  - JF646_intensity: raw JF646 intensity at the detected spot
  - mNG2_intensity: raw mNeonGreen2 intensity at the detected spot
  - file_name: source image name
  - JF646_intensity-norm_factor: JF646 normalization factor corresponding to the median JF646 intensity at the random spots within the given ROI
  - JF646_intensity-norm: normalized JF646 intensity at the detected spot, calculated by dividing the raw intensity by the corresponding normalization factor
  - mNG2_intensity-norm_factor: mNG2 normalization factor (as above)
  - mNG2_intensity-norm: normalized mNG2 intensity at the detected spot (as above)
  - JF549_intensity-norm_factor: JF549 normalization factor (as above)
  - JF549_intensity-norm: normalized JF549 intensity at the detected spot (as above)

ORF1-561-all_random_spots-NORM.csv

- Output data matrix from the spot detection algorithm run on the ORF1-Halo and ORF1-mNG2 colocalization data, with intensity information for the randomly assigned intracellular spots, with the same columns as above
