# Supplementary material for: Condensation of LINE-1 is critical for retrotransposition
Source: eLife. 2023 Apr 28;12:e82991. doi: 10.7554/eLife.82991 (PMC10202459; doi:10.7554/eLife.82991)
Supplement: Figure 1—source data 3. — Output data matrices from the spot detection algorithm run on the ORF1-Halo and reporter L1 RNA FISH colocalization data, with intensity information for the detected Halo+ spots and randomized cytoplasmic ‘Halo’ spots, as well as detected RNA+ spots and randomized cytoplasmic ‘RNA’ spots; associated with Figure 1G. [file elife-82991-fig1-data3.zip › Figure 1-Source Data 3/Figure 1-Source Data 3 README.docx]

Figure 1-Source Data 3

ORF1-all_called_spots-NORM_CYT.csv

- Output data matrix from the spot detection algorithm run on the ORF1-Halo and reporter L1 RNA FISH colocalization data, with intensity information for the detected cytoplasmic Halo+ spots, including intensities in the Halo channel and the RNA channel, with the following columns:
  - plane (z): Z plane of the detected spot
  - row (y): y coordinate of the detected spot
  - col (x): x coordinate of the detected spot
  - radius: radius of the detected spot
  - roi: image name or ROI name in which the spot was detected
  - nuclei_ch_intensity: DAPI intensity at the detected spot
  - Halo_intensity: raw Halo-JF549 intensity at the detected spot
  - RNA_intensity: raw 640-nm RNA intensity at the detected spot
  - location: cytoplasmic localization determined by nuclear stain intensity at the spot as described in the Methods
  - file_name: source image name
  - Halo_intensity-norm: normalized Halo intensity at the detected spot, calculated by dividing the raw intensity by the corresponding normalization factor (median Halo intensity at the random spots within the given ROI)
  - RNA_intensity-norm: normalized RNA intensity at the detected spot (as above)

ORF1-all_random_spots-NORM-CYT.csv

- Output data matrix from the spot detection algorithm run on the ORF1-Halo and reporter L1 RNA FISH colocalization data, with intensity information for the randomly assigned cytoplasmic “Halo” spots, with the same columns as above

RNA-all_spots-NORM_CYT.csv

- Output data matrix from the spot detection algorithm run on the ORF1-Halo and reporter L1 RNA FISH colocalization data, with intensity information for the detected cytoplasmic RNA spots, with the same columns as above. This data was used to determine a threshold for calling RNA-positive detected Halo spots.

RNA-all_random_spots-NORM-CYT.csv

- Output data matrix from the spot detection algorithm run on the ORF1-Halo and reporter L1 RNA FISH colocalization data, with intensity information for the randomized cytoplasmic “RNA” spots, with the same columns as above. This data was used to determine a threshold for calling RNA-positive detected Halo spots.
