# Supplementary material for: Condensation of LINE-1 is critical for retrotransposition
Source: eLife. 2023 Apr 28;12:e82991. doi: 10.7554/eLife.82991 (PMC10202459; doi:10.7554/eLife.82991)
Supplement: Figure 2—source data 1. — Quantification of individual fusion events of WT ORF1p condensates in vitro across RNA concentrations; associated with Figure 2E. [file elife-82991-fig2-data1.zip › Figure 2-Source Data 1 README.docx]

Figure 2-Source Data 1

WT_RNA_ORF1dropletFusionAnalysis.xlsx

- Quantification of individual fusion events of WT ORF1p condensates *in vitro* across RNA concentrations, with each fusion (indicated by “#X”) containing the following columns:
  - Area: total area of the fusing droplets, in µm^2^
  - Mean Int: mean protein channel intensity of the fusing droplets
  - StDev Int: standard deviation of the protein channel intensity of the fusing droplets
  - Major Axis: calculated major axis length of the fusing droplets, in µm
  - Minor Axis: calculated minor axis length of the fusing droplets, in µm
  - Circ: calculated circularity of the fusing droplets
  - AR: calculated aspect ratio of the fusing droplets
  - Time (s): time after the initiation of droplet fusion, in seconds; each fusion was measured every minute for 15 minutes (900 seconds) following initiation of droplet fusion
  - Each fusion has an associated value for:
    - Thresh: protein channel intensity threshold used to identify the fusing droplets
    - Area threshold: area cutoff used in the Analyze Particles function of FIJI to analyze only the droplet fusion of interest
    - Tau: time constant for the fusion calculated from an exponential decay fit to the AR vs Time plot, in seconds (Methods)
    - Fusion Length: a surrogate for fusion size in µm, calculated as the geometric mean diameter of the droplet fusion at time = 0 (Methods)
    - Tau/FusLength: the ratio of fusion time constant tau to fusion length (as above), corresponding to an inverse capillary velocity, in seconds/µm
- The sheets included in the document correspond to the different RNA conditions in which WT ORF1p droplet fusions were analyzed: no RNA (WTprotOnly), 10,000:1 protein:RNA (WT1to10000RNA), 3,000:1 protein:RNA (WT1to3000RNA), and 1,000:1 protein:RNA (WT1to1000RNA)
