# Supplementary material for: Condensation of LINE-1 is critical for retrotransposition
Source: eLife. 2023 Apr 28;12:e82991. doi: 10.7554/eLife.82991 (PMC10202459; doi:10.7554/eLife.82991)
Supplement: Figure 3—source data 1. — Data matrices from the three biological replicates of the puncta counting experiment with wild-type ORF1, ORF1 K3A/K4A, and ORF1 R261A after 6 hours of doxycycline-induced expression in HeLa cells; associated with Figure 3F. [file elife-82991-fig3-data1.zip › Figure 3-Source Data 1 README.docx]

Figure 3-Source Data 1

totalPunctaPerCell_maxZfindMaxima_20210905_HeLaORF1mut_punctaImaging_rep1_Prom75.xlsx

- Data matrix from the first biological replicate of the puncta counting experiment with wild-type ORF1, ORF1 K3A/K4A, and ORF1 R261A after 6 hours of doxycycline-induced expression in HeLa M2 cells, with the following sheets:
  - Summary: summarized puncta counts, where each column represents an ORF1 variant (WT, K3A/K4A, or R261A) and each value represents the total puncta count from a single cellular ROI
  - WT: puncta count values from the cells expressing L1 with wild-type ORF1-Halo, with the following columns
    - FOV: image name
    - Cell: cellular ROI identification number
    - Area: calculated area of the cellular ROI
    - # of Puncta: number of detected puncta in the given cellular ROI
  - K3AK4A: puncta count values from the cells expressing L1 with ORF1-Halo with the K3A/K4A mutations (as above)
  - R261A: puncta count values from the cells expressing L1 with ORF1-Halo with the R261A mutation (as above)

totalPunctaPerCell_maxZfindMaxima_2021009_HeLaORF1mut_punctaImaging_rep2_Prom75.xlsx

- Data matrix from the second biological replicate of the puncta counting experiment with wild-type ORF1, ORF1 K3A/K4A, and ORF1 R261A, as above

totalPunctaPerCell_maxZfindMaxima_2021016_HeLaORF1mut_punctaImaging_rep3_Prom75.xlsx

- Data matrix from the third biological replicate of the puncta counting experiment with wild-type ORF1, ORF1 K3A/K4A, and ORF1 R261A, as above
