# Supplementary material for: Condensation of LINE-1 is critical for retrotransposition
Source: eLife. 2023 Apr 28;12:e82991. doi: 10.7554/eLife.82991 (PMC10202459; doi:10.7554/eLife.82991)
Supplement: Figure 3—source data 2. — FACS output data matrices from the three biological replicates of the 72 hr cellular retrotransposition assay with wild-type ORF1 with and without doxycycline induction, ORF1 K3A/K4A, and ORF1 R261A, all with the GFP-AI retrotransposition reporter in HeLa cells, each with three technical replicates; associated with Figure 3G. [file elife-82991-fig3-data2.zip › Figure 3-Source Data 2 README.docx]

Figure 3-Source Data 2

HeLaORF1mut_72hrDox_retroT_Biorep1.xlsx

- FACS output data matrix from the first biological replicate of the 72-hour cellular retrotransposition experiment with wild-type ORF1 without doxycycline induction (WT No Dox), ORF1 WT (WT Dox), ORF1 K3A/K4A (K3AK4A Dox), and ORF1 R261A (R261A Dox), all with the GFP-AI retrotransposition reporter in HeLa M2 cells, each with three technical replicates, with the following columns:
  - Sample: sample name including ORF1 variant, presence or absence of doxycycline, and technical replicate number
  - EGFP-A %Parent: % of EGFP+ cells, based on a threshold set based on the non-induced WT samples
  - AF647-A %Parent: % of Halo+ cells, based on a threshold set based on the non-induced WT samples

HeLaORF1mut_72hrDoxRetroT_Biorep2.xlsx

- FACS output data matrix from the second biological replicate of the 72-hour cellular retrotransposition experiment with wild-type ORF1 +/- doxycycline, ORF1 K3A/K4A, and ORF1 R261A, as above. The AF647 column is not included because only one biological replicate underwent Halo-JF646 staining prior to FACS.

HeLaORF1mut_72hrDoxRetroT_Biorep3.xlsx

- FACS output data matrix from the third biological replicate of the 72-hour cellular retrotransposition experiment with wild-type ORF1 +/- doxycycline, ORF1 K3A/K4A, and ORF1 R261A, as above.
