# Supplementary material for: Condensation of LINE-1 is critical for retrotransposition
Source: eLife. 2023 Apr 28;12:e82991. doi: 10.7554/eLife.82991 (PMC10202459; doi:10.7554/eLife.82991)
Supplement: Figure 4—source data 1. — Data matrices from the three biological replicates of the puncta counting experiment with wild-type ORF1, ORF1 StammerDel, ORF1 StammerAAA, and ORF1 StammerAEA after 6 hr of doxycycline-induced expression in HeLa cells; associated with Figure 4B. WT ORF1 data is the same as in Figure 3—source data 1, as these samples were all run in parallel. [file elife-82991-fig4-data1.zip › Figure 4-Source Data 1 README.docx]

Figure 4-Source Data 1

totalPunctaPerCell_maxZfindMaxima_20210905_HeLaORF1mut_punctaImaging_rep1_Prom75.xlsx

- Data matrix from the first biological replicate of the puncta counting experiment with wild-type ORF1, ORF1 StammerDel, ORF1 StammerAAA, and ORF1 StammerAEA after 6 hours of doxycycline-induced expression in HeLa M2 cells, with the following sheets:
  - Summary: summarized puncta counts, where each column represents an ORF1 variant (WT, StammerDel, StammerAAA or StammerAEA) and each value represents the total puncta count from a single cellular ROI
  - WT: puncta count values from the cells expressing L1 with wild-type ORF1-Halo, with the following columns
    - FOV: image name
    - Cell: cellular ROI identification number
    - Area: calculated area of the cellular ROI
    - # of Puncta: number of detected puncta in the given cellular ROI
  - StammerDel: puncta count values from the cells expressing L1 with ORF1-Halo with the Stammer Deletion mutation (as above)
  - StammerAAA: puncta count values from the cells expressing L1 with ORF1-Halo with the StammerAAA mutation (as above)
  - StammerAEA: puncta count values from the cells expressing L1 with ORF1-Halo with the StammerAEA mutation (as above)

totalPunctaPerCell_maxZfindMaxima_2021009_HeLaORF1mut_punctaImaging_rep2_Prom75.xlsx

- Data matrix from the second biological replicate of the puncta counting experiment with wild-type ORF1, ORF1 StammerDel, ORF1 StammerAAA and ORF1 StammerAEA, as above

totalPunctaPerCell_maxZfindMaxima_2021016_HeLaORF1mut_punctaImaging_rep3_Prom75.xlsx

- Data matrix from the third biological replicate of the puncta counting experiment with wild-type ORF1, ORF1 StammerDel, ORF1 StammerAAA and ORF1 StammerAEA, as above
