# Supplementary material for: Condensation of LINE-1 is critical for retrotransposition
Source: eLife. 2023 Apr 28;12:e82991. doi: 10.7554/eLife.82991 (PMC10202459; doi:10.7554/eLife.82991)
Supplement: Figure 4—source data 3. — Quantification of individual fusion events of ORF1p K3A/K4A, R261A, StammerAEA, and StammerAAA condensates in vitro across RNA concentrations; associated with Figure 4D. [file elife-82991-fig4-data3.zip › Figure 4-Source Data 3 README.docx]

Figure 4-Source Data 3

K3AK4A_RNA_ORF1dropletFusionAnalysis.xlsx

- Quantification of individual fusion events of ORF1p K3A/K4A condensates *in vitro* across RNA concentrations, with each fusion (indicated by “#X”) containing the following columns:
  - Area: total area of the fusing droplets, in µm^2^
  - Mean Int: mean protein channel intensity of the fusing droplets
  - StDev Int: standard deviation of the protein channel intensity of the fusing droplets
  - Major Axis: calculated major axis length of the fusing droplets, in µm
  - Minor Axis: calculated minor axis length of the fusing droplets, in µm
  - Circ: calculated circularity of the fusing droplets
  - AR: calculated aspect ratio of the fusing droplets
  - Time (s): time after the initiation of droplet fusion, in seconds; each fusion was measured every minute for 15 minutes (900 seconds) following initiation of droplet fusion
  - Each fusion has an associated value for:
    - Thresh: protein channel intensity threshold used to identify the fusing droplets
    - Area threshold: area cutoff used in the Analyze Particles function of FIJI to analyze only the droplet fusion of interest
    - Tau: time constant for the fusion calculated from an exponential decay fit to the AR vs Time plot, in seconds (Methods)
    - Fusion Length: a surrogate for fusion size in µm, calculated as the geometric mean diameter of the droplet fusion at time = 0 (Methods)
    - Tau/FusLength: the ratio of fusion time constant tau to fusion length (as above), corresponding to an inverse capillary velocity, in seconds/µm
- The sheets included in the document correspond to the different RNA conditions in which ORF1p K3A/K4A droplet fusions were analyzed: no RNA (K3AK4AprotOnly), 10,000:1 protein:RNA (K3AK4A1to10000RNA), and 3,000:1 protein:RNA (K3AK4A1to3000RNA)

R261A_RNA_ORF1dropletFusionAnalysis.xlsx

- Quantification of individual fusion events of ORF1p R261A condensates *in vitro* across RNA concentrations, as above

StammerAEA_RNA_ORF1dropletFusionAnalysis.xlsx

- Quantification of individual fusion events of ORF1p StammerAEA condensates *in vitro* across RNA concentrations, as above

StammerAAA_RNA_ORF1dropletFusionAnalysis.xlsx

- Quantification of individual fusion events of ORF1p StammerAAA condensates *in vitro* across RNA concentrations, as above. Only one page is included (the “no RNA” condition), since too few fusion events occurred in the RNA addition conditions for quantification.
